# The Application of Digital Technologies and Artificial Intelligence in Healthcare: An Overview on Nutrition Assessment

**DOI:** 10.3390/diseases11030097

**Published:** 2023-07-13

**Authors:** Alessia Salinari, Michele Machì, Yasmany Armas Diaz, Danila Cianciosi, Zexiu Qi, Bei Yang, Maria Soledad Ferreiro Cotorruelo, Santos Gracia Villar, Luis Alonso Dzul Lopez, Maurizio Battino, Francesca Giampieri

**Affiliations:** 1 Department of Clinical Sciences, Faculty of Medicine, Polytechnic University of Marche, 60131 Ancona, Italy; s1095688@studenti.univpm.it (A.S.); s1102624@studenti.univpm.it (M.M.); y.armas@pm.univpm.it (Y.A.D.); d.cianciosi@staff.univpm.it (D.C.); s1114074@pm.univpm.it (Z.Q.); s1114207@pm.univpm.it (B.Y.); 2 Direzione Medica Ospedaliera, Azienda Ospedaliera-Universitaria delle Marche, 60126 Ancona, Italy; mariasoledad.ferreirocotorruelo@ospedaliriuniti.marche.it; 3 Research Group on Food, Nutritional Biochemistry and Health, Universidad Europea del Atlántico, 39011 Santander, Spain; santos.gracia@uneatlantico.es (S.G.V.); luis.dzul@unini.edu.mx (L.A.D.L.); 4 Department of Projects, Universidad Internacional Iberoamericana, Campeche 24560, Mexico; 5 Department of Extension, Universidad Internacional do Cuanza, Cuito P.O. Box 841, Angola; 6 Department of Projects, Universidad Internacional Iberoamericana, Arecibo, PR 00613, USA; 7 International Research Center for Food Nutrition and Safety, Jiangsu University, Zhenjiang 212013, China

**Keywords:** deep learning, machine learning, mobile health applications, natural language processing, wearable trackers devices

## Abstract

In the last decade, artificial intelligence (AI) and AI-mediated technologies have undergone rapid evolution in healthcare and medicine, from apps to computer software able to analyze medical images, robotic surgery and advanced data storage system. The main aim of the present commentary is to briefly describe the evolution of AI and its applications in healthcare, particularly in nutrition and clinical biochemistry. Indeed, AI is revealing itself to be an important tool in clinical nutrition by using telematic means to self-monitor various health metrics, including blood glucose levels, body weight, heart rate, fat percentage, blood pressure, activity tracking and calorie intake trackers. In particular, the application of the most common digital technologies used in the field of nutrition as well as the employment of AI in the management of diabetes and obesity, two of the most common nutrition-related pathologies worldwide, will be presented.

## 1. Introduction

Nowadays, artificial intelligence (AI) is widely used in many human activities, starting from single individual to factories and companies. According to the modern dictionary definition, AI can be defined as “The theory and development of computer systems able to perform tasks normally requiring human intelligence, such as visual perception, speech recognition, decision-making, and translation between languages” [1]. The main general goal of developing AI is to produce machines or software that could simulate human activities and reasoning, including the capacity to identify images, understand language, solve problems and make decisions learning by error. Depending on how much AI imitates human reasoning, it can be distinguished into three basic categories: “strong AI” that strongly simulates human logical reasoning and behavior; “weak AI” systems that perform fewer human brain activities and the “in-between systems”, those that are inspired by human reasoning. The “in-between systems” do not reproduce human reasoning perfectly but use it as a model guide [2].

In healthcare, the main applications of AI are multiple and are commonly adopted for four different purposes: (i) for improving the treatment of diseases, by increasing the therapeutic efficacy; (ii) for the prediction of diseases, by making, for example, diagnosis at early stages; (iii) for the care and medication of patients, by managing medical records or drug delivery and development, among others; and finally (iv) for monitoring patients in real-time, through customizable early warning scores and regular patient surveillance [3]. Consequently, AI may exert a positive impact on the healthcare system, helping professionals in fast diagnosis, personalized medicine, drug design, disease evaluation and monitoring, including diet-related diseases, and allows the collection and interpretation of a large amount of data in a fast and accurate way, giving continuous feedback and avoiding time-consuming procedures and significantly reducing costs.

Despite the integration of digital technologies and AI in both clinical and medical areas, it is not so common in the field of nutrition, even if recently, especially during the COVID-19 pandemic, progress has been made by using both telematic tools to ensure nutrition assessments at a distance and devices that permit patients to self-monitor different health metrics linked to nutrition, such as blood glucose levels, body weight, heart rate, fat percentage, blood pressure, activity tracking, calorie intake tracking, diet composition and quality [4]. In this context, digital technologies and AI can be a promising and helpful tool, especially in chronic diseases that are highly disabling such as diabetes mellitus, obesity and metabolic disorders which are often challenging to handle and can show non-optimal outcomes. Digital technologies and devices can be connected to AI, allowing the prediction, screening and monitoring of treatment processes, thus helping clinicians, patients, and more generally the whole healthcare system. For instance, smartphone mobile systems, one of the most common digital devices, which are able to identify the type of food on a plate and calculate its nutrient and caloric content, have recently been developed; these systems are essential in aiding patients affected by diabetes to make better decisions regarding their food choices. Similarly, other modern technological devices help patients self-monitor blood glucose concentrations. For its part, AI could be involved in the design of new bioactive small molecules that can be used in medicinal chemistry and in many other biological areas or in discovering bioactive sequences, encoded in food proteins, which may modulate, for example, inflammatory markers. Digital devices should be increasingly integrated with AI systems and applied in daily clinical practice, for predicting the risk of disease, optimizing diets or developing personalized nutrition, among others.

The principal goal of the current work is to briefly present the most common digital technologies and the application of AI in the field of nutrition, with a particular focus on the implication of using AI in the diagnosis and management of diabetes and obesity.

## 2. Digital Technologies and Devices in Nutrition

In recent years, digital technologies and devices, such as mobile health applications and wearable trackers, have simplified the collection of different types of data, in a real-time and longitudinal manner, improving the safety and the quality of nutrition support care. Mobile health applications generally use algorithms to ensure a large amount of data are collected for nutrition assessments, in particular biometric values, laboratory data, food intake tracking and macronutrient/micronutrient tracking.

Evidence suggests that these web-based and smartphone apps ensure a better follow-up, data collection, monitoring and care process and improvements in clinical outcomes, including nutrition knowledge and weight control [5,6,7,8]. The patient can easily download the apps and put in personal data, such as food intake, weight, height, hydration, activity, blood glucose and more, enabling them to continuously self-monitor parameters and progress. All the data can be shared with the health provider who can guide the individual to select and correct the nutritional interventions. Not surprisingly, the mobile applications market has been valued at approximately 40 billion dollars; it is predicted to increase even more in the future.

Specifically, the use of mobile apps for controlling health data is expected to grow by approximately 17.7% from 2021 to 2028; in the United States, health-related apps were downloaded by about 58% of mobile phone users and nearly 83% of dieticians use mobile apps in their daily practice [9,10].

In the field of nutrition, many applications for monitoring weight loss and dietary patterns have been extensively used and studied. For example, an important reduction in body mass index (BMI) of approximately 0.43 kg/m2 has been measured in subjects who used weight loss mobile apps, since these mobile tracking apps provided a continuous real-time response to the health interventions that they followed [11]. Additionally, from the point of view of clinicians, the use of mobile apps, together with the dietary recalls, can represent a valid tool in the care process as they evaluate the real dietary pattern followed by patients who usually tend to under-report their consumption. There are different types of diet-focused apps available on the market, depending on their “easy-to-use” modes or their functions; some of these applications allow users to record both physical activities and dietary intake with the possibility to establish specific objectives from time to time, while others may also offer social aspects by directly connecting with other users or interacting with online forums.

Applications can work simultaneously with other wearable trackers for complete assistance. The synergy between mobile apps and wearable trackers can provide estimated an energy expenditure from heart rate. Wearable dietary trackers are a novel device, increasingly used, to inertly register daily data about health and exercise. These devices, like smartwatches, are equipped with sensors that are able to capture image, sound or even emotion; due to the different types of sensors that trackers have, they can collect a quantity of health-related information. For example, on the one hand, some devices use microphones that, analyzing sounds, report chewing and collect data about the quantity of food ingested and can also quantify the number of bites [12]; on the other hand, other kinds of devices may contain microcameras to identify not only the foods ingested but also the portion sizes. For these reasons, algorithms for identifying food, databases with food images and/or databases with portion size information have been recently developed and tested on several devices that are usually used for monitoring food intake [13]. Some examples are represented by the app named “Snap-n-eat”, which is able to estimate food, energy content, and nutrient intake by simply analyzing the images taken directly by the user through the mobile phone [14], or by the app called “Keenoa”, which works in a similar way, but with the addition of sending the nutrient analysis directly to a dietician [15]. Finally, to monitor emotion, devices are usually equipped with sensors (i.e., gyroscope and/or accelerometer) that capture movements, including rotation, lifting or turning of the wrist, in order to detect the number of bites, and can estimate the total caloric intake by using predictive equations. However, it should be highlighted that these motion-based tools must be worn on the hand used for eating.

Some examples of wearable trackers include fitness trackers, which can monitor various health parameters, such as blood oxygen levels, heart rate, stress levels and sleep; these trackers can also analyze physical activities, like running movements and step counting and suggest improvements. Additionally, other digital applications have been designed to synchronize with continuous glucose monitors or with glucometers to immediately measure the concentration of blood glucose.

Importantly, it must be underlined that these wearable sensors are still in development and that further work is needed to address and clarify some important aspects, including algorithms that are able to discriminate between foods (solid vs. liquid) or more precisely identify food volumes and portions.

## 3. Principles of Artificial Intelligence

**Figure 1:**
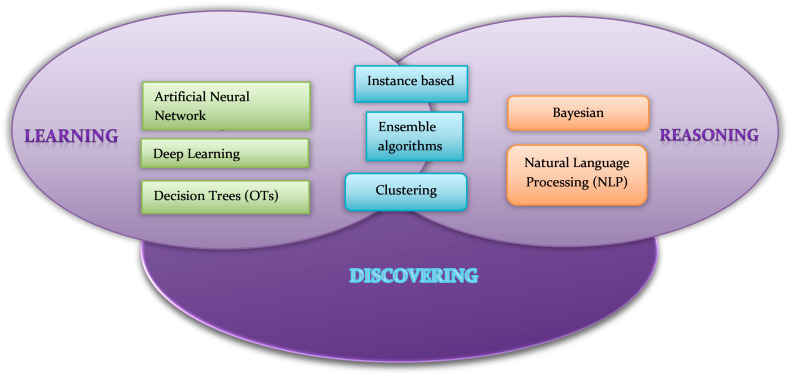
A schematic representation of the most commonly known AI methods.

Machine learning (ML), deep learning (DL) and natural language processing (NLP) are the three most common types of AI techniques [16] (Figure 1).

ML, a subset of AI, shares several similarities with the traditional statistical approach. It is able to discriminate between correct and incorrect categorizations and is oriented towards prediction, through the use of variable algorithms; it includes supervised and unsupervised learning. Supervised learning uses algorithms such as random forests (RF), decision trees (DT), k-nearest neighbor (KNN) or support vector machines (SVMs), to improve predictions (especially in classification or regression) by finding relationships among variables. Unsupervised learning uses algorithms that investigate hidden/natural patterns or relations within the data (especially in clustering, extraction and visualization) without any pre-existing labels [17,18,19,20,21,22].

ML is particularly helpful in processing dietary patterns, macronutrient and food intake data with the use of algorithms like RF, SVM or KNN. For example, ML has been applied to predict malnutrition in children under 5 years and to calculate the risk factors for overweight or obesity in prematurely born children [23,24]. KNN and RF algorithms are able to calculate, on the basis of dietary patterns, long-term cardiometabolic risk by using standard statistical models [25]. Similarly, RF algorithms are being used to improve the cardiovascular mortality risk prediction [26].

On the contrary, DL, a subset of ML that deals with creating self-learning systems, is based on a neural network of algorithms, without the need to process structured data; in fact, a categorization from the outside is not necessary [27]. It is the system itself that identifies the distinctive characteristics in the data and controls whether the classifications need to be modified. In addition, unlike ML, which works with a controllable database and is used for simple routine tasks, DL requires more than 1 million data (e.g., texts, images, social media) to be able to provide desirable results. For this reason, its fields of application are more complex and less used [28].

Finally, NLP, usually used for text analysis, for translation and for speech recognition, can answer questions, paraphrase or detect meaning and context [29]; it is especially used to collect and organize information from medical files, blogs and social media [18].

## 4. Artificial Intelligence in the Management of Nutrition-Related Diseases

Recently, AI has become prominent as a powerful tool that aims to break down the “gold standard” lifestyle of people. People tend to increasingly procrastinate and underestimate their health problems, such as diabetes, obesity and other chronic and non-communicable disorders which, if ignored, can cause permanent damage. New technology systems can participate in this process by allowing real-time access and delivery to treatment, helping overcome these barriers through the use of smartphones, websites, video calls, social media and systems based on algorithms of medium or high complexity. Therefore, AI can be really helpful in disease management, in order to control its progression and prevent complications. Although dozens of AI-based medical devices using AI/ML technology exist and are especially used in the field of radiology, oncology and cardiology, the devices approved and used in the field of nutrition-related diseases are still scarce.

### 4.1. Artificial Intelligence in Diabetes

Diabetes is a complex metabolic condition characterized by high levels of blood glucose and serious clinical complications, whose prevalence is expected to rise worldwide from 537 million in 2021 to 783 million in 2045. The classification of diabetes identifies three main types: type 1, type 2 and gestational type, with type 2 > 90% of the total prevalence [30].

In general, for the diagnosis and treatment of diabetes, the clinical application of AI can be divided into four main sectors: (i) retinal screening; (ii) support for clinical diagnosis; (iii) management tools; and (iv) risk evaluation [31]. Briefly, regarding the first group, AI is already being used to screen and diagnose retinopathy, as demonstrated, for example, by the recently developed algorithm based on deep ML with high specificity and sensitivity for identifying diabetic retinopathy from the fundus images of adult patients [32]. Consequently, using AI in diabetes can increase the prevention, detection and prompt treatment of this common complication, also integrating the continuous monitoring of parameters that can instantly indicate blood glucose fluctuation with new generation glucometers. The second group includes systems that send data collected from continuous glucose monitoring to a cloud server and utilizes AI to remotely decide and suggest the appropriate adjustment for insulin dose; the physicians can then evaluate the suggestion and, if necessary, alert patients [31]. The third group includes tools that help patients to manage their disease themselves, and through which AI collects their data and possibly notifies the physicians to check and ameliorate the control of patient blood glucose. For example, in the Guardian Connect System, AI predicts hypoglycemia 1 h in advance on the basis of data received from continuous glucose monitoring and warning the patient who can take, for example, glucose tablets in time [31]. Finally, the last group involves ML technologies that are able to identify subjects at high risk of developing diabetes, even if ML does not currently outperform the conventional statistical models already employed to calculate the risk of diabetes onset, so that more studies are needed in this regard [31].

AI technologies can be applied for the management of the different types of diabetes. For example, regarding type 1 diabetes, a new generation of closed-loop (CL) systems, based on AI/ML algorithms that are able to continuously evaluate plasma glucose and insulin levels as well as deliver appropriate doses of insulin, has been recently developed; these systems enable the instant prediction of both hyperglycemic and hypoglycemic fluctuation [33]. The best-known systems used in type 1 diabetes include the Cambridge Simulator, the UVA-Padova simulator and the ABBA system, that are all important tools for evaluating insulin absorption, levels and effects on target cells, glucose uptake, availability and levels, prandial carbohydrate absorption and for delivering the most appropriate dose of insulin [34,35,36,37]. In young/adolescent patients affected by type 1 diabetes, the use of these CL insulin delivery systems has been associated with: (i) a reduction in the occurrence of hypoglycemia without increasing the glucose excursions related to meals [38]; (ii) an improvement in the time spent in the range of 70–180 mg/dL, a decrease in the time under (<70 mg/dL) and above (>180 mg/dL) the range and a reduction in overnight hypoglycemia [39] and (iii) a decrease in the levels of glycated hemoglobin (HbA1c) and a concomitant increase in the time spent in the range [40]. Altogether, these results suggest the ability of these systems to improve glycemic control in young patients.

Regarding type 2 diabetes, different AI technologies have been proposed to improve the management protocols, the tracking of patient outcomes and daily-life support for the therapies [41,42,43]; some algorithms have also been developed to help physicians select the appropriate medication [44] as well as to aid patients in attending regular doctor visits [45]. These tools comprise mainly multiple ML techniques (bagging, Bayesian decision trees and SVM) [46], k-means clustering analysis [47,48] and NLP methods [49]. All these studies reported a general improvement of diabetic conditions and patient life quality, including a decrease in the levels of HbA1c levels, an improvement of diabetes awareness and an enhancement of patients’ skills in managing the diabetic disease.

AI could also be useful in pregnancy with diagnosed diabetes. Available technologies, like continuous subcutaneous insulin infusion or automated insulin delivery systems, have been found to ameliorate outcomes in pregnant users [50]. For example, in gestational diabetes mellitus patients AI tools are being used for weight-management counseling [51] or for clinical control (collecting biometric data, physical activity and food intake) through mobile apps or a web support system [52,53], suggesting a general improvement in patient outcomes.

Finally, AI can also be applied in pre-diabetic conditions, where its use, essentially based on a decision support system with reinforcement learning algorithms, has been correlated with a reduction in the HbA1c level and weight, with a concomitant increase in physical activity [54,55]. If prolonged for a long time, the application of AI could decrease the onset and progression of diabetes in this high-risk population.

### 4.2. Artificial Intelligence in Obesity

Obesity is a clinical condition with a high prevalence and multifactorial etiology characterized by an excessive increase in body weight, mainly caused by the increase in adipose tissue that negatively affects the state of health [56]. Genetic predisposition has an incision level of 20–25%, while the remaining percentage depends on individual, behavioral, socioeconomic and psychological factors that can be partly modified by adopting a healthy lifestyle and healthy eating habits, such as the abandonment of a sedentary life and a careful choice in the consumption of meals. For its progressive expansion, especially in the western world, this nutritional disorder is defined by the World Health Organization as a “global epidemic” [57]. Nowadays, obesity is no longer considered a problem of the developed world, because, thanks to the social, cultural and economic progress that occurred towards the second half of the last century, people have established a secondary relationship with food. They pay more attention to low cost and to quantity rather than to the quality of the raw material, with an increasing trend shifted towards processed foods compared to fresh foods such as fruits and vegetables. In general, women present a higher rate of obesity than men, who conversely have a higher rate of being overweight. Obesity may increase the risk of developing common non-communicable diseases, such as cardiovascular and metabolic disorders, as well as some forms of tumor; additionally, it reduces the quality of life and increases the risk of premature death [57].

Obesity is generally defined using a numerical parameter referred to as BMI, which evaluates, in a generic way, the extent to which our weight is above or below the limit threshold; in fact, it does not distinguish between fat mass and lean mass, and the distribution of body fat is not a relevant factor in this case. A subject is defined as obese if their body weight exceeds their ideal weight by 20% [58]. Another parameter used to assess abdominal obesity is the waist circumference/hip circumference ratio (WHR) which should be less than 0.9 in men and 0.85 in women. In the first case, if the ratio is high, we talk about android or “apple” obesity, since the fat is mainly localized at the abdominal level; in the second case, we talk about gynoid or “pear” obesity, where the fat is mainly concentrated in the hips and buttocks. In addition, the circumference of the waist should not exceed 88 cm in women and 102 cm in men, according to the European guidelines [59]. Untreated obesity during the childhood and adolescence period represents a risk factor for the onset in adulthood of a wide variety of pathological conditions (cardiovascular diseases, diabetes, gastrointestinal alterations, metabolic syndrome, atherosclerosis, dyspnea, osteoarthritis, hepatic steatosis, cancer) accompanied by an increased risk of mortality. The frequency of childhood obesity is constantly increasing in populations with a high socio-economic level, with 38.2 million 5-year-old children who are overweight or obese in western countries in 2019 [60].

In order to prevent obesity during childhood and adolescence, ML models, thanks to their predictive power, are becoming potential tools capable of generating very accurate predictions, modeling complex and nonlinear relationships between variables and processing high-dimensional data in this area [61]. One of the advantages of using ML models is to make improvements from a socio-economic point of view to the populations that are at high risk. These predictive models permit a more personalized and economic approach and allow one to classify the risk factors in order of importance, with the aim of designing targeted interventions to prevent and counteract this serious pathology. Thus, through logistic regression, it is possible to quantify the risk of a subject becoming overweight or obese in certain age groups. This has been demonstrated over the years by several studies, such as the work carried out in Germany by Pei et al. [62], or the study by Hammond et al. conducted in New York [63].

To ensure safe prevention, it is necessary to identify in the early stages of age which children are at risk of being overweight. One of the factors that affects the health of the unborn child and childhood obesity is closely linked to the state of health of the mother before and after pregnancy. An unhealthy lifestyle, with the habit of smoking and alcohol, an unbalanced diet with a prevalence of highly processed and unnatural foods, an irregular sleep–wake cycle, a poor level of daily physical activity and short breastfeeding are all determinants associated with the development of obesity in childhood. Until now, very few studies have evaluated the potential application of ML to predict or prevent childhood obesity. For example, in a study carried out on 3121 children, a predictive model was developed considering some determinants described above and collecting scores derived from anthropometric data on weight and height taken during the first 5 years of the children’s life [62]. Family income and parental level of education were also included as covariants. The results of the prediction model were presented as linear regression coefficients (β) for standardized BMI and as an odds ratio (OR) for overweight (yes/no), with the corresponding confidence intervals (CI) of 95%. The results showed that the main risk factors predicting whether a 10-year-old child would be overweight were essentially a high birth weight, high BMI standardized in the first 60–64 months of life (5 years of life), parental education, family income and maternal smoking status during pregnancy. Thus, children with excessive weight at both birth and 5 years of age have a greater chance of being overweight at the age of 10; this possibility is aggravated by maternal smoking during pregnancy, low family income and a low level of parental education. In addition, the study found that children who came from a family with a high level of parental education were less likely to be overweight, reducing this risk at the tenth year of life [62].

Unlike predictive approaches that rely on traditional statistical methods, ML models allow greater completeness in data collection at a low cost. For example, in a recent study, data from electronic health records (EHRs) were collected in order to prevent the onset of obesity during critical periods of child development, from pre-pregnancy to 2 years of life [63]. The EHR data, based on 3449 children, covered both those related to the child and those related to the mother, such as race, nationality, ethnicity, country of origin, home address, languages spoken, vital signs, drugs taken, analyses carried out by laboratory tests and medical examinations (e.g., diabetes mellitus in pregnancy, diabetes mellitus without complications, hypertension in pregnancy, complications at the first year of age). To predict childhood obesity, 156 analyses were carried out using the LASSO logistic regression model, a random forest and a gradient boosting classifier, through which the median value of the BMI was normalized and the classification techniques forecast the dichotomous outcome of obesity status: obese/non-obese were obtained. Results found that the main risk factors for the development of obesity in the first 5 years are an elevated weight and BMI during the first 2 years of life. In fact, by studying the different individual characteristics, the BMI and the weight for length Z-score (average 19–24 months) and the BMI and the weight for length Z-score (latest available reading) were predominantly associated with the outcomes of obesity at the age of 5 for both female and male subjects [63]. These results confirmed that EHR and ML models are useful tools to prevent obesity in those children who have all the high-risk variables. Therefore, through ML algorithms it is possible to identify high-risk children who are likely to develop obesity and, in this sense, could be specifically selected for future interventions.

Depending on the availability of data, it is possible to ensure not only prediction or prevention, but also the monitoring and treatment of obese subjects. If the availability of data is limited, sensors, her and smartphone apps can be used for monitoring. In the latter case, for example, a study was conducted on 69 adult men randomly divided into 2 groups (standard group and mobile group) and treated for 12 months [64]. Volunteers belonging to the standard group, and subjects belonging to the mobile group received personalized digital assistance with coaching calls (twice a week for 6 months), to register the consumption of food, weight and physical activity. The participants of the mobile group lost about 3.9 kg more than those belonging to the standard group, highlighting the fact that the combination of mobile technology and the addition of telephone coaching can represent a solution to improve weight loss in obese subjects.

Conversely, for a larger dataset, ML methods provide more sophisticated models to predict obesity-related risks and outcomes. Besides linear and logistic regression, other common ML methods include decision tree analysis, artificial neural networks, deep learning and reinforcement learning, all applied for obesity management. Logistic regression and decision tree analysis are exclusively used for classification, while artificial neural networks and deep learning can not only allow classification, but can also be applied to predict a continuous variable. For example, decision tree analysis is a predictive algorithm that uses a combination of categorical input data and continuous values with the aim of assigning samples to specific classes. They have been used in several studies to predict which patients, among those who had undergone bariatric surgery, had a higher chance of long-term post-operative success [65] and to predict childhood obesity in high-risk age groups [66]. 

Finally, the artificial neural network is a mathematical representation of the architecture of the human neurological system and is used for the prediction of both numerical and categorical continuous data. Artificial neural networks are self-adaptive models that can adapt their data architecture without specifying the form, both functional and distributive [67]. Studies show that the use of artificial neural networks is a tool capable of making predictions on the success of bariatric surgery to which obese patients are subjected [65,68]. Even today its use in the field of obesity is rather limited, it could become an important resource to improve and enhance the chances of success in long-term weight loss.

## 5. Conclusions and Future Directions

In conclusion, the advances made in the development of AI have led to more and more sophisticated applications and tools that enable the performance of outcome-based research and assess trends in care delivery. AI has started to be used to evaluate biological pathways, to make diagnosis, to predict medical outcomes through devices like mobile phones, smartwatches and others, leading to a more personalized collection of data and feedback from customers in real time [69]. AI systems could create the possibility of improving nutrition assessments, collecting and evaluating data about dietary intake and providing diets that fit one’s personal context and behaviors. Also, AI devices may permit the creation of practical and precise feedback that is essential in clinical nutrition.

In the future, the adoption of AI could open the avenue for the development of personalized nutrition and the enhancement of the support care system that could lead to better health at the individual and national levels. Indeed, there is growing awareness that, at a nutritional level, the approaches suggested for the general population do not always address the needs of each individual, since each person may respond in a different way to food nutrients, because of different factors, including age, gender, microbiota, genetics, metabolism and lifestyle habits, among others [70,71]. Therefore, from the individual point of view, AI technologies could speed up the objective to reach good health well-being, by making precise customized dietary recommendations and encouraging the development of both predictive and preventive guidelines to promote health and manage diseases in a better way. From the point of view of the national healthcare system, AI could help physicians in selecting the appropriate therapy, in adjusting the dose in time and whenever necessary, as well as in identifying patients who need more exhaustive or urgent examinations from those who have good metabolic control; additionally, AI could lighten the work load of physicians, by reducing the time spent in face-to-face visits, as well as decrease the waiting list in medical centers, also leading to a reduction in general healthcare costs. Therefore, digital technologies and AI are promising tools for health promotion and disease prevention and management, but some issues still need to be addressed, mainly those connected with patient privacy; thus, close collaboration between healthcare institutions and research is desirable in the future.

## Data Availability

No new data were created or analyzed in this study.
